# FLOWERING LOCUS T2 Promotes Shoot Apex Development and Restricts Internode Elongation *via* the 13-Hydroxylation Gibberellin Biosynthesis Pathway in Poplar

**DOI:** 10.3389/fpls.2021.814195

**Published:** 2022-02-03

**Authors:** Daniela Gómez-Soto, Isabel Allona, Mariano Perales

**Affiliations:** ^1^Centro de Biotecnología y Genómica de Plantas, Universidad Politécnica de Madrid, Centro Nacional Instituto de Investigación y Tecnología Agraria y Alimentaria, CNINIA (CSIC), Madrid, Spain; ^2^Departamento de Biotecnología-Biología Vegetal, Escuela Técnica Superior de Ingeniería Agronómica, Alimentaria y de Biosistemas, Universidad Politécnica de Madrid, Madrid, Spain

**Keywords:** poplar, Flowering Locus T (FT), dormancy-growth cycles, gibberellin, GA1, shoot apex development, GA 13-hydroxylation pathway, internode elongation

## Abstract

The adaptation and survival of boreal and temperate perennials relies on the precise demarcation of the growing season. Seasonal growth and development are defined by day length and temperature signals. Under long-day conditions in spring, poplar FLOWERING LOCUS T2 (FT2) systemically induces shoot growth. In contrast, FT2 downregulation induced by autumnal short days triggers growth cessation and bud set. However, the molecular role of FT2 in local and long-range signaling is not entirely understood. In this study, the CRISPR/Cas9 editing tool was used to generate FT2 loss of function lines of hybrid poplar. Results indicate that FT2 is essential to promote shoot apex development and restrict internode elongation under conditions of long days. The application of bioactive gibberellins (GAs) to apical buds in FT2 loss of function lines was able to rescue bud set. Expression analysis of GA sensing and metabolic genes and hormone quantification revealed that FT2 boosts the 13-hydroxylation branch of the GA biosynthesis pathway in the shoot apex. Paclobutrazol treatment of WT leaves led to limited internode growth in the stem elongation zone. In mature leaves, FT2 was found to control the GA 13-hydroxylation pathway by increasing *GA2ox1* and reducing *GA3ox2* expression, causing reduced GA1 levels. We here show that in poplar, the FT2 signal promotes shoot apex development and restricts internode elongation through the GA 13-hydroxylation pathway.

## Introduction

Growth and development in perennial trees are coordinated by synchronization between plant internal events and environmental cues. Photoperiod and temperature are the main factors that regulate multiple processes such as dormancy growth reactivation or flowering. In response to a short-day (SD) photoperiod, poplar trees stop their growth and develop buds to protect meristems anticipating colder temperatures. Once a chilling requirement has been fulfilled, the cold period required to release buds from dormancy (Rohde et al., 2000), warmer temperatures and long-day (LD) conditions trigger bud break and growth reinitiation (Weiser, 1970; Cooke et al., 2012; Singh et al., 2020). The identification of poplar orthologs of FLOWERING LOCUS T (FT), FT1, and FT2, as flowering and seasonal mediators of perennial growth–dormancy cycles was an important breakthrough in defining the molecular framework that orchestrates these transitions (Bohlenius et al., 2006; Hsu et al., 2011). However, the physiological and molecular functions of FT2 during the poplar growing season remain unclear.

In multiple plant species, FT has been identified as an essential component of the signaling module that controls photoperiod-induced flowering, tuberization, nodulation, and annual growth–dormancy cycles (Koornneef et al., 1991; Kobayashi et al., 1999; Bohlenius et al., 2006; Tamaki et al., 2007; Navarro et al., 2011; Wang et al., 2021). The Arabidopsis *FT* gene expresses companion cells in leaf phloem under specific LD conditions (Corbesier et al., 2007; Jaeger and Wigge, 2007; Mathieu et al., 2007; Chen et al., 2018). To trigger flowering, the mobile FT protein travels from the leaf to the shoot meristem (Corbesier et al., 2007; Jaeger and Wigge, 2007; Andrés and Coupland, 2012). Several studies in perennials have shown that FT orthologs are necessary to control photoperiod-induced growth cessation and bud set during the vegetative stage and to promote flowering onset (Bohlenius et al., 2006; Hsu et al., 2011; Karlgren et al., 2013). Poplar has *FT1* and *FT2* paralogs that show specific temporal and spatial expressions (Hsu et al., 2011). *FT1* transcription is activated in the shoot apex during winter (Hsu et al., 2011; Pin and Nilsson, 2012; Gómez-Soto et al., 2021). Gain of function studies in poplar has revealed that FT1 coordinates reproductive onset (Hsu et al., 2011). In response to LD and warm temperatures, *FT2* is expressed in the leaves, showing a peak of mRNA accumulation at dusk (Bohlenius et al., 2006; Hsu et al., 2011; Triozzi et al., 2018). Analysis of poplar FT2 RNA interference (RNAi) has indicated that FT2 knockdown reduces vegetative growth and accelerates growth cessation and bud set (Hsu et al., 2011). Through transcriptional profiling after transient induction of *FT1* or *FT2*, a divergent yet uncharacterized molecular network has been described in poplar (Hsu et al., 2011).

The regulation of poplar FT2 function resembles that of Arabidopsis FT (Bohlenius et al., 2006; Hsu et al., 2006). *FT2* transcription is day-length sensitive (Bohlenius et al., 2006). The coincidence between day or night length and the expression of poplar orthologs of the Arabidopsis circadian-controlled factors, *CONSTANTS*, *GIGANTEA*, *CYCLIN DOFs*, and *LATE HYPOCOTHYL 2*, sets the daily window of *FT2* expression in the leaf (Bohlenius et al., 2006; Ding et al., 2018; Ramos-Sánchez et al., 2019). The poplar shoot vegetative annual growth–dormancy cycle requires long-range FT signaling from the leaf to shoot apex (Miskolczi et al., 2019). FT2 forms a transcriptional complex with poplar orthologs of Arabidopsis FLOWERING LOCUS D LIKE 1 and 2 (FDL1 and FDL2) (Tylewicz et al., 2015). This FT2-FDL1 complex activates *Like-APETALA 1* (*LAP1*) and its downstream target gene *AINTEGUMETA LIKE 1* (*AIL-1*), promoting shoot apex development. Downregulation of *LAP1* or *AIL1* reduces shoot meristem cell division leading to poplar growth cessation under a SD regime (Karlberg et al., 2011; Azeez et al., 2014).

Different phytohormones have been attributed a role in coordinating plant development (Santner et al., 2009). Gibberellins (GAs) are growth regulators and promoters of developmental shifts such as flowering, branching, cambial stem cell differentiation, seed dormancy release, annual growth–dormancy cycles, and other processes (Blázquez et al., 1998; Kucera et al., 2005; Rinne et al., 2011; Felipo-Benavent et al., 2018; Katyayini et al., 2020).

The biosynthesis of GA takes place in three stages: (1) ent-kaurene is synthesized in plastids and is catalyzed by ent-copalyl diphosphate synthase (CPS) and ent-kaurene synthase (KS). The transcription of these enzymes depends on developmental stage and is cell type specific; (2) at the ER membrane, ent-kaurene is converted into GA_12_ in a reaction catalyzed by ent-kaurene oxidase (KO) and ent-kaurenoic acid oxidase (KAO), and these genes are expressed in all tissues examined. There is an additional step in which GA_12_ can be converted into GA_53_ by GA 13-oxidase, so from this point, two parallel biosynthetic pathways may proceed, non-13-hydroxylation directly from GA_12_ or 13-hydroxylation from the GA_53_ precursor (Yamaguchi, 2008; Hedden, 2020); and (3) GA_12_ and/or GA_53_ are converted into GA intermediates and finally bioactive GAs. These steps take place in the cytosol and are catalyzed by GA 20-oxidases (GA20ox) and GA 3-oxidases (GA3ox), whose expression shows tissue-specific patterns (Hedden and Phillips, 2000; Yamaguchi and Kamiya, 2000). The final products of the non-13-hydroxylation pathway are GA_4_ and GA_7_ and of the 13-hydroxylation pathway are GA_1_, GA_3_, GA_5_, and GA_6_ (Yamaguchi, 2008; Hedden, 2020). Bioactive GA binds GIBBERELLIN-INSENSITIVE DWARF (GID) F-box protein causing a conformational switch that triggers degradation of DELLA proteins *via* the ubiquitin-26S proteasome system. DELLA is a subfamily of the GRAS family of transcription factors that are negative regulators of GA signaling (Nelson and Steber, 2018). Precursors and bioactive GA levels are regulated by their rate of biosynthesis and deactivation. This last process is catalyzed by GA 2-oxidases (GA2ox) which inactivate GA products (Thomas et al., 1999). In poplar, GA biosynthesis and deactivation enzymes have been extensively studied in multiple tissues and developmental states, and their genes also show specific expression patterns (Gou et al., 2011; Katyayini et al., 2020). In this tree, GA is required for the release of winter dormancy and the reactivation of shoot apical growth (Rinne et al., 2011; Eriksson et al., 2015). Bioactive GAs regulate shoot branching whereas the dormant state requires high levels of GA_3_/GA_6_ in axillary buds, which reduces GA_1_/GA_4_. The accumulation of GA_1_/GA_4_ correlates with axillary bud outgrowth (Katyayini et al., 2020). Transgenic poplar shows the downregulation of biosynthesis or sensing genes show reduced branching and internode and leaf lengths (Busov et al., 2003; Zawaski et al., 2011). Constitutive overactivation of GA catabolism through *GA2ox1* gives rise to dwarfed trees (Busov et al., 2003). Moreover, GA promotes cambial stem cell differentiation to produce xylem (Bhalerao and Fischer, 2017). Hence, in poplar, shoot development and seasonal shifts are GA-dependent.

FT and GA interaction has been described during plant development. It has been well established that FT and GA signaling are required to control Arabidopsis flowering through independent pathways. FT and GA promote this transition in response to LD and SD, respectively (Eriksson et al., 2006; Hisamatsu and King, 2008). However, GA depletion in Arabidopsis shoot apical meristem delays flowering under conditions of LD, indicating that active GA signaling is required for flowering (Porri et al., 2012). In rice, the FT ortholog RFT1 induces stem elongation increasing GA sensitivity in the stem (Gómez-Ariza et al., 2019). In poplar, FT1 and GA have been shown to promote dormancy release and bud break. Chilling treatment activates *FT* transcription and some GA biosynthesis and deactivation genes prior to bud break (Rinne et al., 2011; Gómez-Soto et al., 2021). Poplar overexpressing *AtGA20ox1* with increased GA_4_ and GA_1_ levels shows delayed growth cessation under SD conditions, whereas *FT2* is downregulated. Accordingly, a dual control mechanism by parallel pathways has been proposed for dormancy onset (Eriksson et al., 2015). Grafting experiments have shown that both FT protein and an unknown GA signal can move from leaf to shoot to maintain shoot apex growth (Miskolczi et al., 2019). Moreover, *FT1* overexpressing poplar shows lower expression levels of *GA2ox8* in shoot apex under LD conditions, suggesting FT1 and GA metabolism interaction (Miskolczi et al., 2019). However, it remains to be clarified whether FT1 controls GA metabolism locally or systemically.

In this work, we used the CRISPR/Cas9 editing tool to generate FT2 loss of function lines in hybrid poplar. Our phenotypic analyses reveal that FT2 loss of function plants set buds and show elongated internodes under growth promoting conditions. Through transcriptional and metabolic analyses, we detected interaction between FT2 and the GA 13-hydroxylation pathway. Accordingly, we propose a dual role of poplar FT2 in promoting shoot apex development and restricting internode elongation *via* control of this pathway and GA1 levels.

## Materials and Methods

### Plant Material and Growth Conditions

Hybrid poplar *Populus tremula* × *P. alba INRA clone 717 1B4* was used as the wild-type control and for plant transformation. Poplars were cultured *in vitro* following procedures reported earlier (Gómez-Soto et al., 2021) and transferred to 3.5-L pots containing blond peat, pH 4.5, kept in a growth chamber under controlled conditions (22°C, LD 16-h light/8-h dark, 65% relative humidity, and 150 μmol light intensity). Just once, 2 weeks after transplantation, the plants were fertilized with a solution of 1 g/L Peters Professional 20–20–20 (Everris International, Geldermalsen, Netherlands).

### Generation of CRISPR/Cas9 Construct

To generate the CRISPR/Cas9 construct targeting the *FT2* gene, we followed the procedures described by Jacobs et al. (2015). The sgRNA within the first exon of the hybrid poplar *FT2* gene was obtained from a predesigned SNP-free gRNA dataset.^1^ The DNA fragment of the U6 promoter and scaffold were amplified using Phusion High-Fidelity DNA Polymerase (Thermo Fisher Scientific, MA, United States) and purified from the gel using NucleoSpin Gel and PCR Clean-up (Macherey-Nagel, PA, United States). The oligonucleotides used to generate the construct are listed in Supplementary Table 1. Briefly, *Swa*I- and *Spe*I-digested p201N Cas9 plasmid (Jacobs et al., 2015) was mixed with the U6 single-strand oligonucleotide (containing the sgRNA sequence) and scaffold DNA fragments and ligated using NEBuilder^®^ HiFi DNA Assembly Master Mix (New England Biolabs, MA, United States). The reaction product, shown in Supplementary Figure 1A, was transferred to *E. coli* DH5α competent cells (Thermo Fisher Scientific, MA, United States) following procedures reported by Inoue et al. (1990). Positive bacterial clones containing the CRISPR/Cas9-FT2 construct were identified by colony PCR using the primers Ubi3p218R and ISCeIR (Supplementary Table 1). The correct assembly and sequence of the CRISPR/Cas9-FT2 construct was confirmed by DNA sequencing using Ubi3p218R primer.

### Plant Transformation, Transformant Selection, and Genotyping

The CRISPR/Cas9-FT2 construct was transferred to Agrobacterium tumefaciens strain GV3101/pMP90 (Konczl and Schell, 1986). Hybrid poplar was transformed *via* an Agrobacterium-mediated protocol described previously (Gallardo et al., 1999) with the modification described recently by Gómez-Soto et al. (2021). Transformed hybrid poplar explants were selected under 50 mg/mL of kanamycin-containing medium until whole plantlets were regenerated. Twenty-five independent hybrid poplar kanamycin-resistant lines were regenerated. The specific *FT2* genome edition was investigated in these lines by PCR amplification of the *FT2* and *FT1* gene fragments using specific primers flanking the sgRNA site (Supplementary Table 1), cloning the fragment into NZY-A PCR cloning kit (NZYtech, Lisbon, Portugal) and sequencing up to 15 independent clones per line. The presence of T-DNA insert in these lines was detected by PCR using the specific primers 35Sfwd (located at the end of the 35S promoter) and Cas9rev (located at the beginning of the *Cas9* gene) and amplified about 528 bp of T-DNA (Supplementary Figure 1B). Sequences were aligned using the ClustalW multiple alignment tool in the BioEdit Sequence Alignment Editor 7.0 (Hall, 1999). Four out of the twenty-five lines (lines #8, #10, #20, and #23) appeared edited in the *FT2* gene causing gene loss of function. Noticeably, shoot growth in the *in vitro* cultured hybrid poplar CRISPR/Cas9-FT2 edited lines #8, #10, #20, and #23 was as slow as in controls.

### Plant Phenotyping

Once control plants had reached bud stage 3, bud score phenotyping of *ft2-8*, *ft2-10*, and wild-type plants was initiated. Bud set progression was graded by scoring from stage 3 (fully growing apex) to stage 0 (fully formed apical bud) according to Rohde et al. (2010). The assay was repeated two times with similar results (*n* = 6).

Internode lengths were measured in 2-week-old *ft2-8*, *ft2-10*, and wild-type plants in the stem elongation zone (from internode 1–2 to 7–8). As an example, a representative internode 1–2 of wild-type and *ft2-8* plants is indicated in Supplementary Figure 2. The assay was repeated two times with similar results (*n* = 6).

### Gibberellin and Paclobutrazol Treatments, and Plant Phenotyping

For the GA treatments, 50 μL of a solution of GA3 10 μM (Alfa Aesar, Lancashire, United Kingdom) or GA 4 + 7 10 μM (Duchefa Biochemie, Haarlem, Netherlands) was applied to the apex of each *ft2-8* and *ft2-10* plant. Plants were treated every 2 days for 18 days. As control treatment, a water solution in an equivalent volume of ethanol (GA dissolvent) was used. To measure the timing of bud break after hormone treatment, bud score was quantified from stage 0 (fully formed apical bud) to stage 5 (fully growing apex) according to Rohde et al. (2010). The assay was repeated two times with similar results (*n* = 5).

For PACLOBUTRAZOL (PAC) treatment, a 0 or 100 μM solution of PAC in water (Duchefa Biochemie, Haarlem, Netherlands) was used to spray the leaves of 4-week-old wild-type plants. This treatment was performed every 2 days for 15 days. On the 15th day of treatment, internode length was measured in the stem elongation zone (from internodes 1–2 to 7–8). The assay was repeated two times with similar results (*n* = 5).

### Tissue Sampling and Gene Expression Analysis

For comparative gene expression analysis of *ft2-8* and wild-type plants, samples of shoot apices and leaves were collected 15 h after the light turned on, zeitgeber time 15 (ZT15), when FT2 shows a mRNA peak under conditions of 16-h light/8-h dark and temperature 22°C (Ramos-Sánchez et al., 2019). Six shoot apices and six mature leaves per plant were hand-cut and immediately frozen on dry ice. Two biological replicates were collected. The bulk samples were stored at –80°C until grinding for RNA extraction. Total RNA was isolated following procedures described in Gómez-Soto et al. (2021). Complementary DNA was synthesized using the Maxima First Strand cDNA Synthesis Kit for RT-qPCR with dsDNase (Thermo Fisher Scientific, MA, United States) according to the manufacturer’s instructions. Quantitative real-time PCR (RT-qPCR) analyses were carried out in a Roche LightCycler 480 II instrument (Roche Diagnostics, Barcelona, España), and numerical values were obtained using the relative quantification method reported by Livak and Schmittgen (2001). Results were normalized with respect to expression of the UBQ7 gene (Pettengill et al., 2012). A complete list of the primers used in the RT-qPCR analysis is provided in Supplementary Table 1.

### Gibberellin Quantification

Thoroughly ground plant tissue (about 100 mg fresh/dry weight) was suspended in 80% methanol–1% acetic acid containing internal standards and mixed by shaking for 1 h at 4°C. The extract was kept at −20°C overnight and then centrifuged, and the supernatant dried in a vacuum evaporator. The dry residue was dissolved in 1% acetic acid and passed through a reverse phase column (HLB Oasis 30 mg, Waters), as described by Seo et al. (2011). The final residues were dried and dissolved in 5% acetonitrile–1% acetic acid. The hormones were separated by UHPLC on a reverse Accucore C18 column (2.6 μm, 100 mm length; Thermo Fisher Scientific) with a 2–55% acetonitrile gradient containing 0.05% acetic acid, at 400 μL/min over 21 min.

The hormones were analyzed in a Q-Exactive mass spectrometer (Orbitrap detector; Thermo Fisher Scientific) by targeted selected ion monitoring (tSIM; capillary temperature 300°C, S-lens RF level 70, resolution 70.000) and electrospray ionization (spray voltage 3.0 kV, heater temperature 150°C, sheath gas flow rate 40 μL/min, auxiliary gas flow rate 10 μL/min) in negative mode.

The concentrations of hormones in the extracts were determined using embedded calibration curves and the software packages Xcalibur 4.0 (Thermo Fisher Scientific) and TraceFinder 4.1 SP1 (Thermo Fisher Scientific). As internal standards for quantifying the different plant hormones, deuterium-labeled hormones were used (purchased from OlChemim Ltd., Olomouc, Czech Republic).

### Statistical Analysis

One-way ANOVA was used for pairwise comparisons. For multiple group comparisons, we also performed Tukey’s *post hoc* test. All statistical tests were performed using the program IBM SPSS statistics for Windows, version 20. Significance was set at *p* < 0.05.

## Results

### FLOWERING LOCUS T2 Is Essential to Promote Shoot Apex Development and Restrict Internode Elongation in Response to a Long-Day Photoperiod

To investigate the role of FT2 as a promoter of shoot development, we generated hybrid poplar FT2 loss of function lines. Two genome-edited hybrid poplar lines were identified (designated *ft2-8* and *ft2-10*) which show a predicted non-functional *FT2* gene because of a frameshift created in the first exon that translates into a truncated protein (Supplementary Figure 3A). Due to the high DNA sequence homology between *FT2* and *FT1*, we confirmed that the *FT1* gene and its protein were unmodified (Supplementary Figure 3B).

**FIGURE 1:**
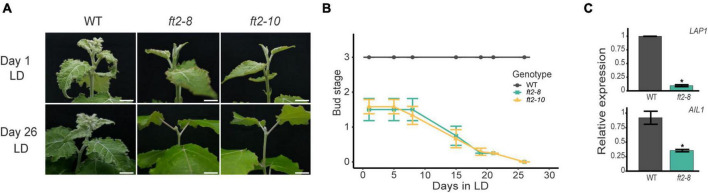
FLOWERING LOCUS T2 (FT2) is essential for shoot development under long-day (LD) conditions. **(A)** Images of the shoot apex of the wild type (WT) and FT2 loss of function lines *ft2-8* and *ft2-10*. *Top panels* show pictures taken on Day 1 when the WT plants had reached stage 3. *Bottom panels* show pictures taken on Day 26, when *ft2-8* and *ft2-10* reached stage 0. Scale bar = 1 cm. **(B)** Line plot showing bud scores for WT, *ft2-8*, and *ft2-10* plants collected until *ft2-8* and *ft2-10* reached stage 0. **(C)** Relative mRNA levels of *LAP1* and *AIL1* genes obtained from WT and *ft2-8* apical buds. *Ubiquitin7* was used as the housekeeping gene. Plotted values and error bars are fold-change means ± SD recorded in two biological replicates. Asterisks (*) represent significant differences assessed by one-way ANOVA (*p* < 0.05) between genotypes.

To test the behavior of *ft2-8* and *ft2-10* under LD conditions in the growth chamber, we transferred *ft2-8*, *ft2-10*, and wild type (WT) *in vitro* grown plantlets to soil-filled pots and scored their apical shoot development. After 2 weeks, WT plantlets went into full leaf production featuring three young leaves on each apex (bud stage 3), whereas *ft2-8* and *ft2-10* apices had only 1 or 2 leaves (bud stage 1.5). At this point, we monitored shoot development for approximately 30 days (Figures 1A,B). During the following days, WT apices continued to undergo full leaf production, yet *ft2-8* and *ft2-10* apices showed a rapid decline in producing leaves and set buds on Day 26 (Figures 1A,B). FT2 loss of function plants were unable to resume growth under growth promoting conditions. In addition, WT plants bore three lateral branches on average yet there were no branches on the *ft2-8* and *ft2-10* plants (Supplementary Figure 4). Hence, in response to LD, *ft2-8* and *ft2-10* plants are incapable of both sustained shoot apex development and axillary bud outgrowth.

**FIGURE 2:**
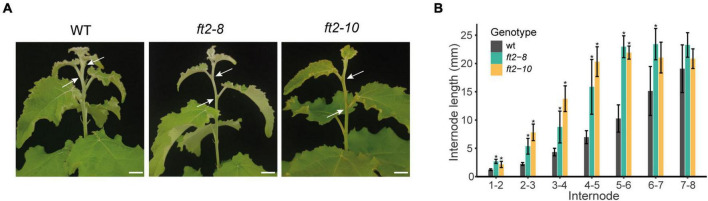
FT2 limits internode growth in the stem elongation zone under LD conditions. **(A)** Images of the stem elongation zone in the WT and FT2 loss of function lines *ft2-8* and *ft2-10*. Arrows indicate internode 4–5. Scale bar = 1 cm. **(B)** Bar plot showing internode lengths in the stem elongation zone of the WT and FT2 loss of function lines *ft2-8* and *ft2-10*. Values represent mean internode lengths recorded in *n* = 5 plants. Significant differences between genotypes were assessed by Tukey’s test, *p < 0.05.

In FT2 loss of function plants, we also observed significantly longer internodes in the stem elongation zone (top 6 new-formed internodes) relative to the WT plants, whereas older internode distances were similar (Figures 2A,B). This trend continued until shoot growth cessation and bud set in the *ft2-8* and *ft2-10* lines (Figures 2A,B). These results indicate that FT2 is essential to restrict internode growth in the stem elongation zone under LD conditions.

As the growth behavior of our *ft2-8* and *ft2-10* lines was indistinguishable, thereafter we only used the *ft2-8* line for our relative gene expression and hormone quantification studies.

The activities of the photoperiod response transcription factors *Like-APETALA1* (*LAP1*) and *AINTEGUMENTA-like 1* (*AIL1*) are required to promote shoot apex development under LD conditions (Karlberg et al., 2011; Azeez et al., 2014). Thus, we examined *LAP1* and *AIL1* expression levels in the shoot apex of FT2 loss of function and WT plants. Our qRT-PCR data confirmed *LAP1* and *AIL1* downregulation in *ft2-8*, supporting the notion that FT2 loss of function plants undergo growth cessation (Figure 1C). Collectively, these results indicate that FT2 is essential to sustain shoot apex development and promote *LAP1* and *AIL1* expression under a LD photoperiod regime.

### Bioactive Gibberellins Are Able to Restore Shoot Apex Development in FLOWERING LOCUS T2 Loss of Function Lines

**FIGURE 3:**
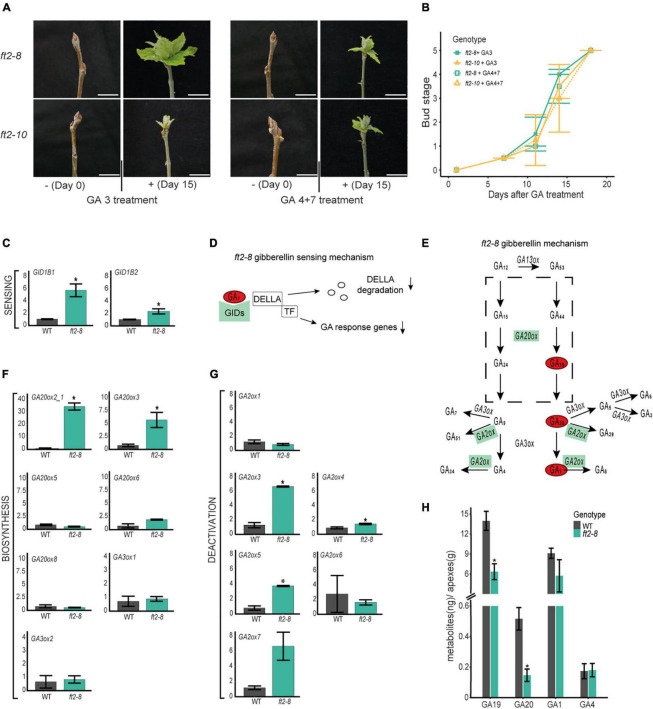
FT2 promotes the gibberellin (GA) biosynthesis 13-hydroxylation pathway branch in the shoot apex. **(A)** Images of apical buds of WT, *ft2-8*, and *ft2-10* lines treated with 0 μM and 10 μM of GA 3 or GA 4 + 7. Pictures were taken on Day 0 and Day 15 of GA treatment. Scale bar = 2 cm. **(B)** Line plot showing bud scores for WT, *ft2-8*, and *ft2-10* plants collected weekly from Day 0 of GA treatment until buds reached stage 5. **(C)** Bar plot showing relative mRNA levels of GA sensing genes *GID1B1* and *GID1B2* in *ft2-8* and WT shoot apex. **(D)** Schematic representation of the GA-GIDs-DELLA molecular model (Nelson and Steber, 2018). High *GID* expression and lower GA_1_ levels might reduce GA responses in the shoot apex. **(E)** Schematic representation of GA metabolism. Green rectangle denotes genes found upregulated in *ft-8* compared to WT shoot apex. Red oval shows precursor and bioactive GA found at lower levels in *ft-8* than WT shoot apex. **(F,G)** Bar plot showing relative mRNA levels in *ft2-8* and WT shoot apex of **(F)** the GA biosynthesis genes *GA20ox* and *GA3ox*, and **(G)** GA deactivated genes *GA2ox*. **(H)** Bar plot showing the quantification of precursors and bioactive GA in WT and *ft2-8* shoot apex. The Y-axis scale shows metabolites (ng)/apex (g), and the X-axis indicates the precursor or bioactive GA measured, that is, GA_19_, GA_20_, GA_1_, or GA_4_. **(C,F,G)** Plotted values and error bars are fold-change means ± SD recorded in two biological replicates. *Ubiquitin7* was used as the housekeeping gene. Asterisks (*) represent significant differences between genotypes assessed by one-way ANOVA (*p* < 0.05).

Gibberellins are required for shoot development in poplar (Busov et al., 2003; Rinne et al., 2011; Eriksson et al., 2015; Miskolczi et al., 2019). To assess whether the exogenous addition of bioactive GAs would enable the resumption of shoot apex development, we treated mature buds of *ft2-8* and *ft2-10* plants with 10 μM GA 3 or GA 4 + 7 once every 2 days for 18 days. Buds were monitored and quantified. After 7 days, GA-treated plants showed larger greener buds (Figures 3A,B). In the following days, buds opened in *ft2-8* and *ft2-10* plants and leaf development resumed until the full-growth stage was reached on Day 18 (Figures 3A,B). Thus, the exogenous application of GA 3 or GA 4 + 7 to apical buds reactivates shoot apex development, suggesting limited GA activity and/or response in the shoot apex of FT2 loss of function plants.

### FLOWERING LOCUS T2 Boosts the 13-Hydroxylation Route of the Gibberellin Biosynthesis Pathway in the Shoot Apex

We then went on to investigate whether GA pathway and sensing gene transcription are affected in the apex of FT2 loss of function plants. To this end, comparative expression analysis of GA sensing, biosynthesis, and catabolic genes was conducted on dissected shoot apices of *ft2-8* and WT plants. First, we analyzed the GA receptors *GIBBERELLINS INSENSITIVE GENES (GIDs)*. Our qRT-PCR analysis revealed significant *GID1B1* and *GID1B2* upregulation in *ft2-8* plants (Figures 3C,D) and no differences in *GID1A1* and *GID1A2* mRNA accumulation between *ft2-8* and WT plantlets (Supplementary Figure 5). The higher *GID* levels that detected in FT2 loss of function plants suggest that these plants might be depleted of bioactive GA in the shoot apex as it was shown in *Arabidopsis* (Middleton et al., 2012).

To examine the GA biosynthesis pathway, we assayed *GIBBERELLINS 20 OXIDASE (GA20ox)* genes involved in GA biosynthesis, from the precursor GA_12_ to the step immediately before synthesizing bioactive GA (GA_9_ and GA_20_) (Eriksson et al., 2000; Hedden and Phillips, 2000). *GA20ox2* and *GA20ox3* were found significantly upregulated in the apex of FT2 loss of function lines compared to WT (Figures 3E,F). In addition to this, we examined expression levels of *GIBBERELLINS 3 OXIDASE GA3ox1* and *GA3ox2*, which catalyze the final step to bioactive GAs (Israelsson et al., 2004). Our results indicate no differences between *GA3ox1* and *GA3ox2* mRNA accumulation in *ft2-8* plants compared to WT (Figures 3E,F). Based on these results, we can rule out the downregulation of GA biosynthesis genes in the shoot apex of FT2 loss of function plants.

Next, we examined GA catabolism by analyzing the expression of *GIBBERELLINS 2 OXIDASE* genes (*GA2ox*) (Gou et al., 2011). Our data revealed the significant upregulation of *GA2ox3*, *GA2ox4*, and *GA2ox5* in *ft2-8* plants in the apex compared to WT (Figures 3E,G). Activation of these *GA2ox* genes suggests local depletion of bioactive GAs in the shoot apex of FT2 loss of function plants. Together, these results suggest that FT2 is required to maintain optimal mRNA level of GA receptors and GA metabolic pathway genes in the shoot apex under LD conditions.

In another series of experiments, shoot apex GA hormone levels were quantified in *ft2-8* and WT plants (Figure 3H and Supplementary Table 2). Levels of GA_1_ in the shoot apex were found to be some 50 times higher compared to GA_4_, thus suggesting the prevalence of the 13-hydroxylation branch of the GA pathway in poplar. Remarkably, *ft2-8* plants showed an almost 40% reduction of GA_1_ levels compared to WT, supporting our prediction of bioactive GA depletion in our comparative transcription expression studies (Figure 3H and Supplementary Table 2). In addition to this, *ft2-8* plants showed 2- and 4-fold reductions in the GA biosynthesis precursors GA_19_ and GA_20_, respectively, than WT plants, suggesting that the low presence of precursors could lessen GA biosynthesis. These results indicate that FT2 loss of function leads to reduced levels of precursors and active GA_1_ in the shoot apex.

### Paclobutrazol-Treated Wild Type Leaves Feature Limited Internode Lengths

**FIGURE 4:**
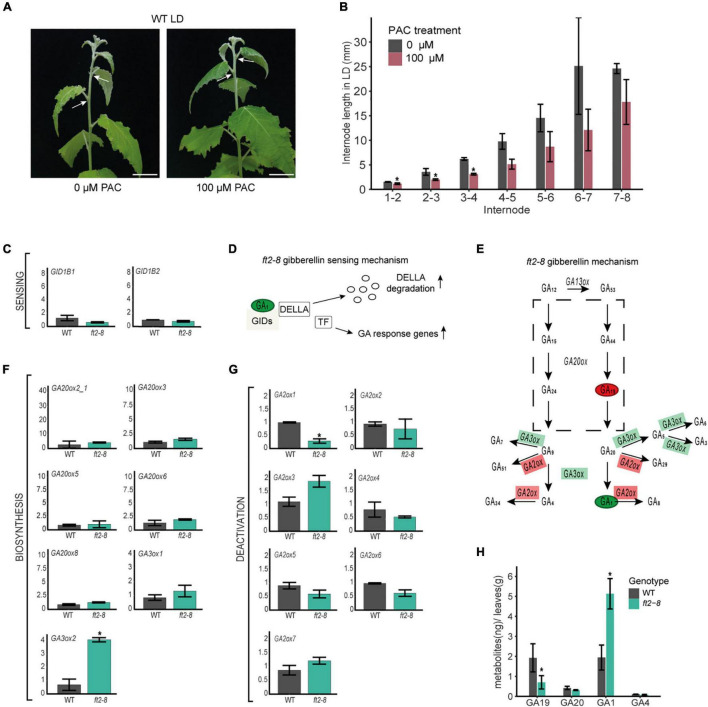
FT2 restricts the GA 13-hydroxylation pathway branch in mature leaf tissue. **(A)** Images of WT shoot treated with 0 or 100 μM of PAC for 15 days under LD conditions. White arrows indicate internode 5–6. Scale bar = 2 cm. **(B)** Bar plot showing internode length (mm) in the stem elongation zone of WT plants treated with 0 μM and 100 μM of PAC for 15 days under LD conditions. Plotted values and error bars are fold-change means ± SD recorded in two biological replicates. Asterisks (*) represent significant differences assessed by one-way ANOVA (*p* < 0.05) between PAC treatments. **(C)** Bar plot showing relative mRNA levels of the GA sensing genes *GID1B1* and *GID1B2* in WT and *ft2-8* leaf tissue. Plotted values and error bars are fold-change means ± SD recorded in three biological replicates. Asterisks (*) represent significant differences between PAC treatments assessed by one-way ANOVA (*p* < 0.05). **(D)** Schematic representation of the GA-GIDs-DELLA molecular model (Nelson and Steber, 2018). High GA_1_ levels might increase GA responses. **(E)** Schematic representation of GA metabolism. Green and red rectangles denote genes found upregulated and downregulated in *ft-8* compared to WT leaf tissue, respectively. Green and red ovals show precursor and bioactive GA found at higher and lower levels in *ft-8* compared to WT leaves, respectively. **(F,G)** Bar plot showing relative mRNA levels in *ft2-8* and WT leaf tissue. **(F)** The GA biosynthesis genes *GA20ox* and *GA3ox*, and **(G)** GA deactivation genes *GA2ox*. **(H)** Bar plot showing the quantification of precursors and bioactive GA in WT and *ft2-8* shoot apex. The Y-axis scale represents metabolites (ng)/apex (g), and the X-axis indicates the precursor or bioactive GA measured that is, GA_19_, GA_20_, GA_1_ or GA_4_. **(C,F,G)** Plotted values and error bars are fold-change means ± SD recorded in two biological replicates. *Ubiquitin7* was used as the housekeeping gene. Asterisks (*) represent significant differences between genotypes assessed by one-way ANOVA (*p* < 0.05).

Our results show that FT2 limits internode length in the shoot elongation zone (Figures 2A,B). Leaf-induced GA signaling controls internode growth in tobacco and rice (Dayan et al., 2012; Gómez-Ariza et al., 2019). To test whether leaf-derived GA is required for stem elongation in poplar, we treated the leaves of WT plantlets with 0 or 100 μM of the GA biosynthesis inhibitor PAC for 15 days under LD conditions. In response, the stems of PAC-treated plants’ leaves showed significantly reduced internode lengths (Figures 4A,B), whereas shoot apices remained at stage 3 (Supplementary Figure 6). This finding indicates that leaf-derived GA and/or GA signaling are necessary for internode growth in the stem elongation zone.

### FLOWERING LOCUS T2 Controls the Gibberellin 13-Hydroxylation Pathway, Increasing *GA2ox1* and Reducing *GA3ox2* in Mature Leaves

The above results also suggest that the observed internode elongation in FT2 loss of function plants could be due to changes in leaf-derived GA. We therefore examined the expression of GA receptor, biosynthesis, and deactivation genes in mature leaves of *ft2-8* and WT. Remarkably, significant mRNA accumulation was only observed of the biosynthesis *GA3ox2* gene, levels of the deactivating *GA2ox1* gene being reduced (Figures 4C–G and Supplementary Figure 7). Of note, *GA2ox1* is the main GA inactivating gene expressed in poplar leaf tissue (Supplementary Figure 8; Katyayini et al., 2020). The regulation by FT2 of GA3ox2 and GA2ox1 mRNA levels suggests a local increase in bioactive GAs in the mature leaves of FT2 loss of function plants.

Gibberellin hormone levels were also determined in mature leaf tissue in *ft2-8* and WT plants (Figure 4H and Supplementary Table 2). Our results show that GA_1_ is the predominant bioactive GA in the leaf, with levels 2.5 times higher in *ft2-8* than in WT plants, supporting our prediction in the comparative transcription expression studies. Further, we detected significantly lower GA_19_ precursor levels in the *ft2-8* than WT plants. Taken together, these results indicate that FT2 controls the GA 13-hydroxylation pathway and GA_1_ levels in mature leaves, correlating with FT2 control of internode elongation.

## Discussion

### FLOWERING LOCUS T2 Is Essential to Promote Shoot Apex Development by Inducing the Gibberellin 13-Hydroxylation Pathway

Arabidopsis FT knockout plants show a late-flowering phenotype indicating that FT is required but not essential for this developmental transition (Yamaguchi et al., 2005). Functional dissection of poplar FT paralogs through RNA interference has identified a role of FT1 and FT2 in coordinating annual reproductive and vegetative development, respectively (Hsu et al., 2011). The GWAS study identified a single major locus containing the *FT2* gene that could explain 65% of phenotypic variation in poplar bud set transition (Wang et al., 2018). Our results indicate that poplar FT2 is essential to sustain vegetative growth in the shoot apex under LD conditions (Figures 1A–C). In its absence, poplar cannot support shoot apex development and forms mature buds after 26 days under LD conditions (Figures 1A–C) resembling the behavior of SD-grown plants. Accordingly, poplar shoot apex development is fully dependent on the expression of a single gene, *FT2*, and the photoperiod through FT2.

Gibberellin promotes plant development transitions including tree dormancy release (Blázquez et al., 1998; Karlberg et al., 2010). Alteration of the GA biosynthesis pathway in GA20ox antisense plants results in earlier bud set in poplar, and plants overexpressing GA20ox show delayed bud set under SD conditions (Eriksson et al., 2015). Moreover, grafting experiments in GA20ox-overexpressing rootstocks have shown that GA can systemically modulate dormancy entrance, ceasing growth later than in WT rootstocks (Miskolczi et al., 2019). Thus, it has been proposed that GA acts in a parallel pathway to FT2 controlling shoot apex growth and photoperiodic responses (Eriksson et al., 2015). FT and LAP1 could play a mediating role in the transcriptional control of GA metabolism in the shoot apex, as *FT-* and *LAP1* overexpressing plants show the reduced expression of *GA2ox 8-3* under LD conditions (Miskolczi et al., 2019). Our following results support the FT2-dependent nature of GA activity in poplar shoot apex under LD conditions: (a) LD-formed mature buds in FT2 loss of function plants resumed growth after the application of bioactive GA to the apex (Figure 3A); (b) the GA pathway mRNA profile of the shoot apex is specific in FT2 loss of function plants compared to WT (Figures 3C–G); and (c) shoot apex levels of bioactive GA_1_ and 13-hydroxylation pathway precursors of GA_19_ and GA_20_ are lower in FT2 loss of function than WT plants (Figure 3H). Collectively, our data reveal that poplar trees require FT2 to promote GA responses in the shoot apex, maintaining shoot development under LD conditions. Interestingly, specific shoot apical meristem GA activity is also needed for Arabidopsis flowering under LD conditions (Porri et al., 2012). However, in Arabidopsis shoot apex, it is not known whether GA activity is FT-dependent.

### FLOWERING LOCUS T2 Controls Internode Elongation and Tunes Gibberellin_1_ Levels in Mature Leaves

Apart from the conserved role of FT in flowering induction, recent work has shown that the rice *FT* ortholog *RFT1* promotes internode elongation *via* downregulation of the *PREMATURE INTERNODE ELONGATION 1 (PINE 1)* gene (Gómez-Ariza et al., 2019). Downregulation of *PINE1* induces increased GA responsiveness in the stem (Gómez-Ariza et al., 2019). Here, we show that FT2 is required to restrict internode growth in poplar during the vegetative phase (Figures 2A,B). According to this observation, FT ortholog overexpressing poplar and tomato plants show shorter stems than WT during the vegetative phase (Bohlenius et al., 2006; Shalit-Kaneh et al., 2019). Therefore, FT could oppositely modulate stem growth depending on the vegetative growth stage in poplar and tomato, or flowering developmental stage in *Arabidopsis* and rice.

The regulation of internode elongation is dependent on plant levels of bioactive GA_1_ and GA_4_ (Eriksson et al., 2000; Ragni et al., 2011; Dayan et al., 2012). GA2ox-hyperactivated poplar lines show reduced bioactive gibberellins GA_1_ and GA_4_ and reduced internode length (Busov et al., 2003). The accumulation in stems of GA and precursors derived from mature leaves promotes internode growth in tobacco (Dayan et al., 2012). Dayan et al. showed that defoliation induces extremely short internodes in the tobacco stem shoot elongation zone. Our results indicate that inhibition of GA biosynthesis through PAC treatment of mature leaves reduces internode growth in WT poplar plants. Thus, leaf GA biosynthesis is necessary for poplar stem elongation (Figures 4A,B). In contrast, FT2 loss of function plants show longer internodes than WT in the shoot elongation zone (Figures 2A,B) along with elevated GA_1_ levels in mature leaf tissue, whereas GA_4_ levels remain similar or reduced (Figure 4H). Moreover, our data indicate that the 13-hydroxylation pathway is prevalent in mature poplar leaf and shoot apex (Figures 3H, 4H). It is widely accepted that the mature leaf supplies bioactive GA and precursors to promote the growth of aerial growing tissues (Regnault et al., 2015). This means that the higher GA_1_ levels observed here in the leaves of FT2 loss of function plants could promote internode growth. However, this GA_1_ increment was insufficient to support shoot apex development, perhaps because of the reduced plasmodesmata channel connections between the rib and shoot apical meristem in poplar (Ruonala et al., 2008). Collectively, our data indicate that FT2 fine tunes GA_1_ levels in mature leaf tissue through *GA3ox2* and *GA2ox1* genes and suggest that leaf GA_1_ is required for internode elongation during poplar vegetative growth under LD conditions.

The role of FT in the regulation of leaf gene expression has been recently reported in Arabidopsis leaf tissue under a LD photoperiod (Andrés et al., 2020). Andres et al. confirmed that Arabidopsis flowering requires the FT-mediated induction of *SWEET10* expression in leaf companion cells. SWEET10 belongs to a plasma membrane transporter protein family linked to sucrose or GA mobilization (Kanno et al., 2016). Nevertheless, how FT activates *SWEET10* is unknown. Our study also points to FT2-dependent transcriptional control of the GA pathway in leaf tissue under conditions of LD (Figures 4C–G). This transcriptional control of the GA pathway by FT2 needs to be further investigated.

## Conclusion

**FIGURE 5:**
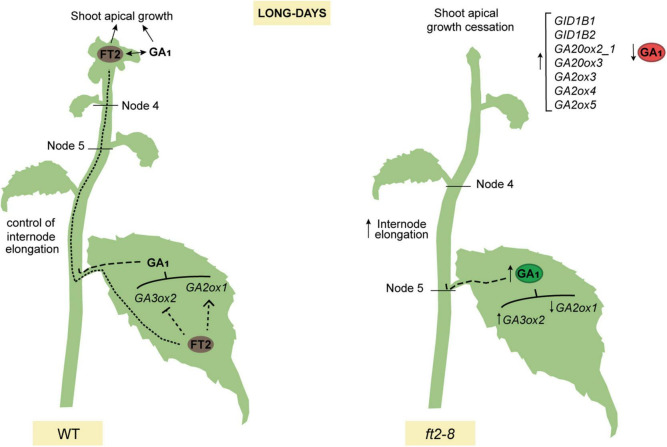
Model showing that poplar FT2 modulates the 13-hydroxylation branch of the GA pathway to promote shoot apex development and restrict internode elongation. In WT plants (*left panel*), FT2 protein is synthesized in the leaf and moves to the shoot apex to promote development. FT2 is required to modulate expression of genes of the 13-hydroxylation branch of the GA pathway and GA_1_. We propose that leaf-derived GA limits internode elongation in stem. In shoot apex, FT2 is essential to modulate the expression of genes of the GA 13-hydroxylation pathway, to maintain levels of GA_19_, GA_20_, and GA_1_, and for shoot apex development. In contrast, in *ft2-8* plants (*right panel*), the lack of FT2 causes an increase in leaf GA_1_ levels, internode elongation, and a decrease in shoot apex GA_1_ levels.

We propose a model whereby FT2 plays a dual role in poplar vegetative growth under LD conditions. FT2 is essential to promote the GA 13-hydroxylation pathway and GA_1_ levels sustaining shoot apex development. Contrarily in leaves, FT2 tunes the GA pathway, limiting GA_1_ production and restricting internode elongation (Figure 5).

## Data Availability Statement

The original contributions presented in the study are included in the article/Supplementary Material, further inquiries can be directed to the corresponding authors.

## Author Contributions

DG-S and MP performed the experiments. DG-S, MP, and IA participated in the design of the experiments and in the discussions described here, wrote the manuscript, contributed to the article, and approved the submitted version.

## Conflict of Interest

The authors declare that the research was conducted in the absence of any commercial or financial relationships that could be construed as a potential conflict of interest.

## Publisher’s Note

All claims expressed in this article are solely those of the authors and do not necessarily represent those of their affiliated organizations, or those of the publisher, the editors and the reviewers. Any product that may be evaluated in this article, or claim that may be made by its manufacturer, is not guaranteed or endorsed by the publisher.

## Footnotes

1
http://aspendb.uga.edu/
