# Supplementary material for: FLOWERING LOCUS T2 Promotes Shoot Apex Development and Restricts Internode Elongation via the 13-Hydroxylation Gibberellin Biosynthesis Pathway in Poplar
Source: Front Plant Sci. 2022 Feb 3;12:814195. doi: 10.3389/fpls.2021.814195 (PMC8853612; doi:10.3389/fpls.2021.814195)
Supplement: Supplementary file 1 [file Data_Sheet_1.pdf]

Figure S1. (A)Squeme showing the CRISPR/Cas9-FT2 construct in p201N vector obtained by SnapGene Viewer (from Insightful Science; available at snapgene.com). (B) PCR Detection of a Cas9 gene fragment in *ft2-8*, *ft2-10*, and wild-type genomic DNA.

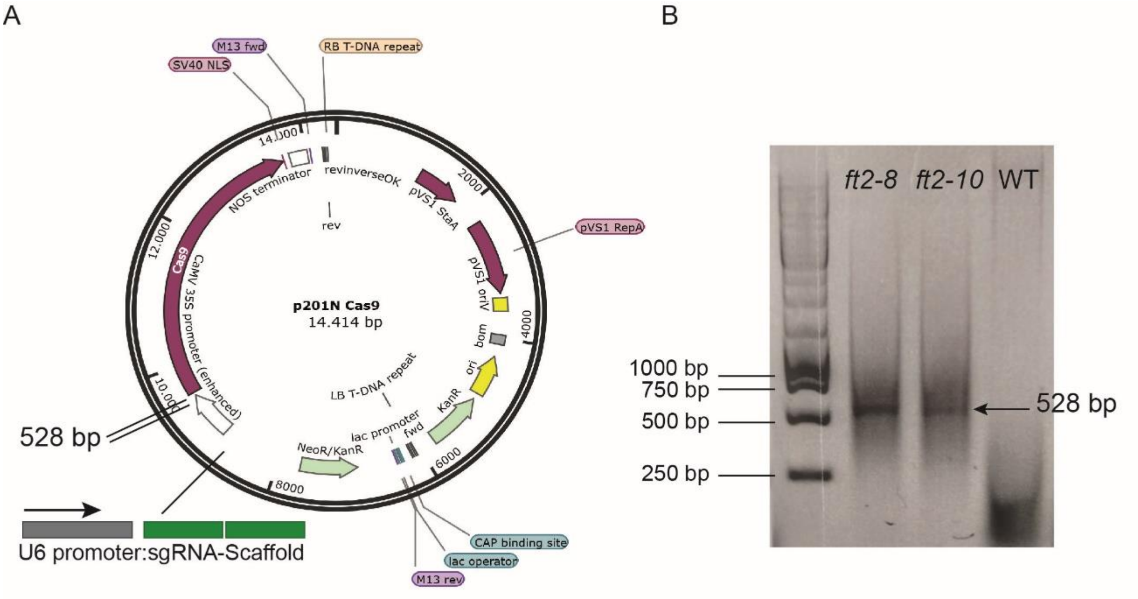

Figure S2. Images depicting the internode measurement approach. Internode 1-2 was established in WT and *ft2-8* apical shoots and photos show both genotypes in front view (left panels) and top view (right panels). Arrows indicate the 1st and 2nd leaf and the internode 1-2. Scale bar = 0.5 cm.

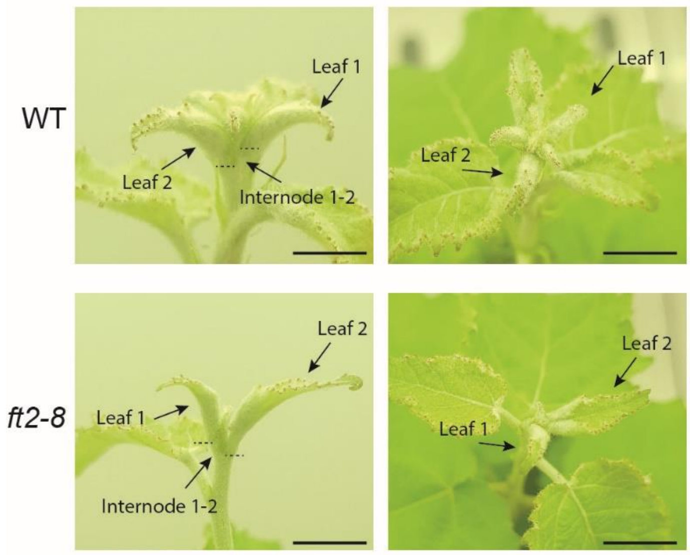

Figure S3. (A)Scheme depicting the structure of *FT2* gene and the localization of the gRNA used for CRISPR/Cas9 edition. The 5'UTR and 3'UTR are shown in grey, exons in green, and introns in black. (A-B) *FT2* and *FT1* DNA sequence (first 93 and 102 nucleotides, respectively) and the predicted protein obtained from, WT, *ft2-8,* and *ft2-10 tremula* and *alba* alleles. Alignments were performed using ClustalW Multiple alignment with a number of 1000 bootstraps. Matching nucleotides among gRNA and *FT2* or *FT1* are indicated in red ink. Insertion or deletion are indicated in blue ink.

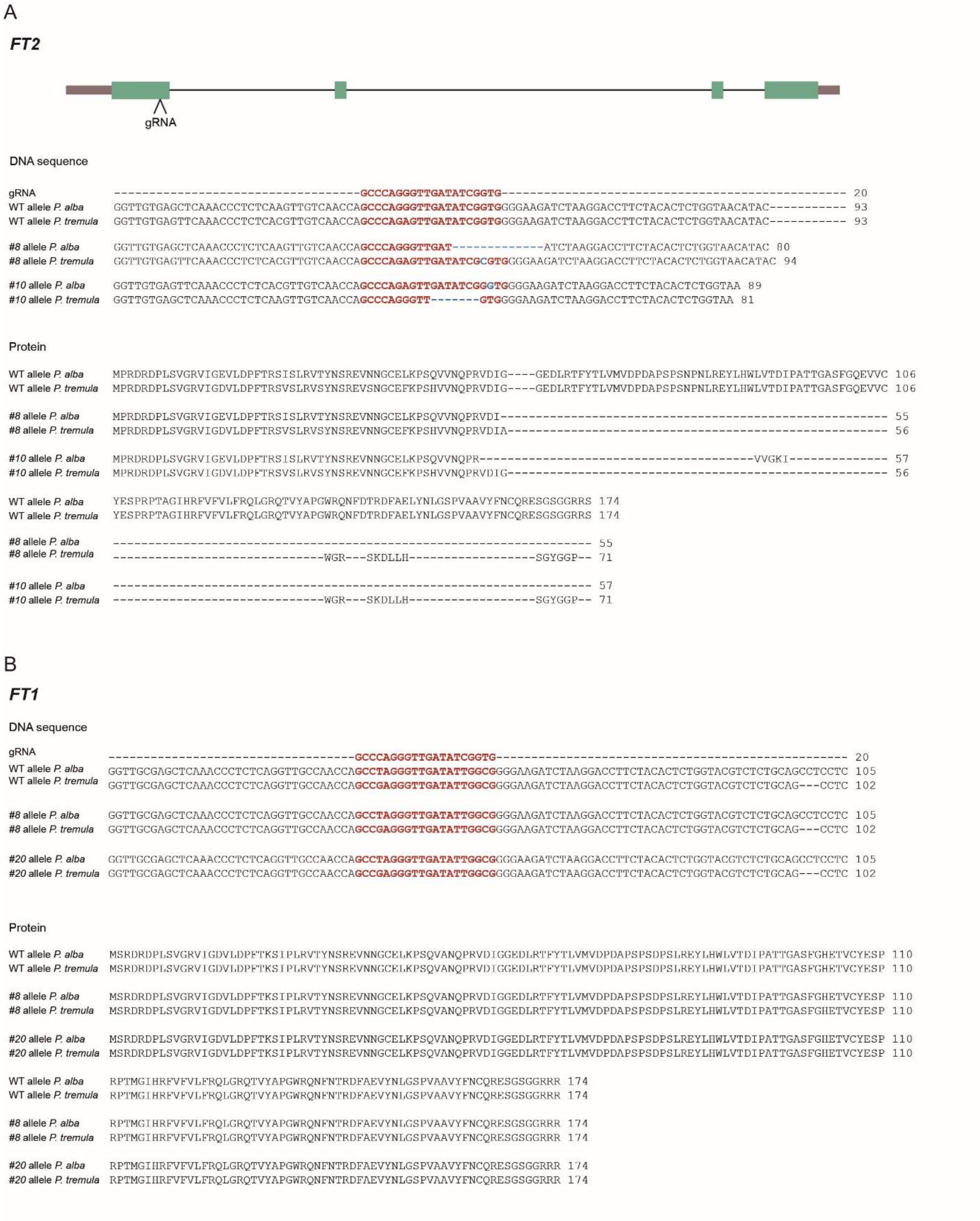

Figure S4. Bar plot showing the quantification branch number of WT, *ft2-8*, and *ft2-10* soil grown plants under LD conditions. The y-axis indicates the number of branches once WT plants reached full growth and the x-axis the genotype analysed.

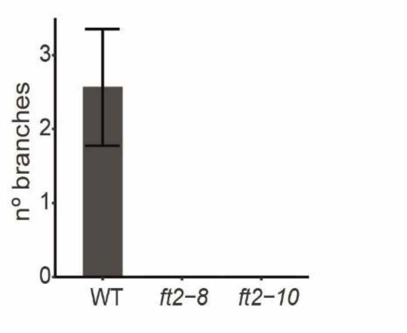

Figure S5. Bar plot showing the relative mRNA level of GA sensing genes *GID1A1* and *GID1A2* in *ft2-8* and WT shoot apex. Plotted values and error bars are fold-change means ± s.d. of two biological replicates. Asterisks (*) represent statistical differences assessed by one-way ANOVA ($p<0.05$). *Ubiquitin7* is used as the housekeeping gene.

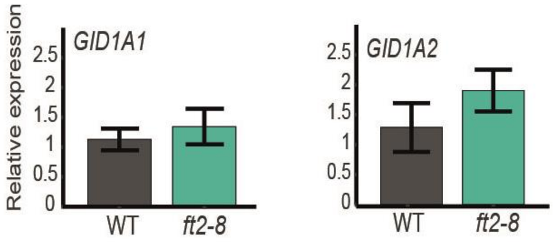

Figure S6. (A-B) Images showing WT shoot apex after 0 µM (A) or 100 µM (B) of PAC treatment for 15 days. Scale bar = 1 cm.

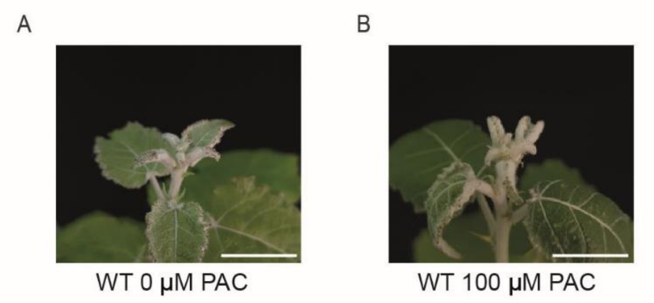

Figure S7. Bar plot showing the relative mRNA level of GA sensing genes *GID1A1* and *GID1A2* in *ft2-8* and WT leaf. Plotted values and error bars are fold-change means ± s.d. of two biological replicates. Asterisks (*) represent statistical differences assessed by one-way ANOVA ($p<0.05$). *Ubiquitin7* is used as the housekeeping gene.

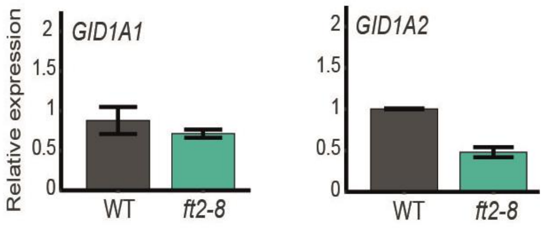

Figure S8. Bar plot showing the relative mRNA level of *GA2ox* genes in WT leaf. Plotted values and error bars are fold-change means ± s.d. of two biological replicates. *Ubiquitin7* is used as the housekeeping gene.

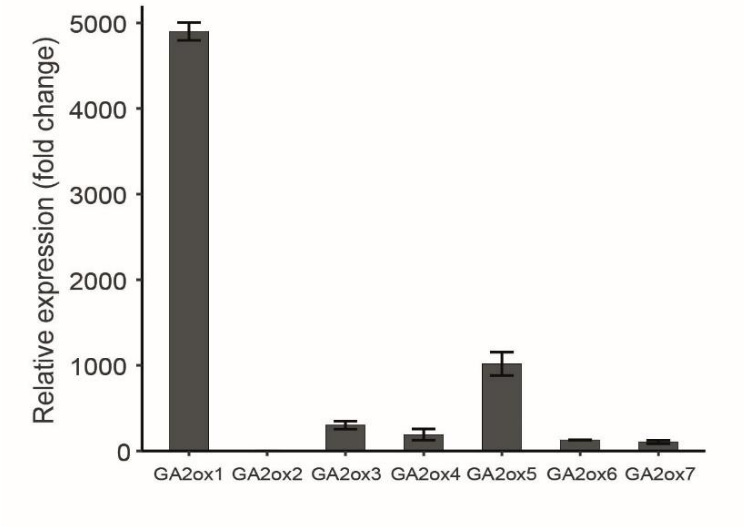

Table S1. List of primers used.

Gene	Gene ID	Primer name	Sequence
U6 promoter	Jacobs et al., 2015	SwaI_MtU6F	GATATTAATCTCTTCGATGAAATTTATGCCTATCTTATATGATCAATGAGG
U6 promoter	Jacobs et al., 2015	MtU6R	AAGCCTACTGGTTCGCTTGAAG
Scaffold	Jacobs et al., 2015	ScaffoldF	GTTTTAGAGCTAGAAATAGCAAGTT

Scaffold	Jacobs et al., 2015	SpeI_ScaffoldR	GTCATGAATTGTAATACGACTCAAAAAAAAGCACCGACTCGGTG
p201	Jacobs et al., 2015	Sequence_p201_Ubi3	ACATGCACCTAATTTCACTAGATGT
FT2	Potri.010G179700	Grna_FT2	TCAAGCGAACCAGTAGGCTT—GCCCAGGGTTGATATCGGTG—GTTTTAGAGCTAGAAATAGC
FT2	Potri.010G179700	Genotype_FT2_fwd	ACCCTCTTAGTGTTGGCCGTGT
FT2	Potri.010G179700	Genotype_FT2_rev	TCACCGCTCATCAGGTTTCAAG
FT1	Potri.008G077700	Genotype_FT1_rev	AAACATGCACGTTTGGCTGC
GA2ox1	Potri.001G378400	GA2ox1_qPCR_fwd	TTCTTCTCATTACCGCTCTCTG
GA2ox1	Potri.001G378400	GA2ox1_qPCR_rev	TCTACCCAGCCCACATCAC
GA2ox2	Potri.002G191900	GA2ox2_qPCR_fwd	GGATGCCTTCCAGGTTTTAACGA
GA2ox2	Potri.002G191900	GA2ox2_qPCR_rev	GCGGAGAGATCCATGCGTTG
GA2ox3	Potri.004G065000	GA2ox3_qPCR_fwd	GGACCTCCTAACCCTTTTGG
GA2ox3	Potri.004G065000	GA2ox3_qPCR_rev	CAGCAGAGCGGAAAATCTGTGG
GA2ox4	Potri.008G101600	GA2ox4_qPCR_fwd	AGGTAGGGTTTGGAGAGCAT
GA2ox4	Potri.008G101600	GA2ox4_qPCR_rev	GGTAGCGGGATCAGGTGTTA
GA2ox5	Potri.010G149700	GA2ox5_qPCR_fwd	GCACCCCCACTTAATGCAAG
GA2ox5	Potri.010G149700	GA2ox5_qPCR_rev	TATCTCCAAGTCGCAGAGCA
GA2ox6	Potri.011G095600	GA2ox6_qPCR_fwd	CAAGCCAGCACTTCAACAGT
GA2ox6	Potri.011G095600	GA2ox6_qPCR_rev	ATTCCTCACATGCCTTAACC
GA2ox7	Potri.014G117300	GA2ox7_qPCR_fwd	TTGCTTGCACGATGGTTTGT
GA2ox7	Potri.014G117300	GA2ox7_qPCR_rev	GCCTCACGCTTTTAAATCTCCC
GA3ox1	Potri.001G176600	GA3ox1_qPCR_fwd	TGGCTCTCCTCTTGAGCATT
GA3ox1	Potri.001G176600	GA3ox1_qPCR_rev	AACCATGTCAACCTCCTTG
GA3ox2	Potri.003G057400	GA3ox2_qPCR_fwd	AACTCCCTATCTCGCTCAATCT
GA3ox2	Potri.003G057400	GA3ox2_qPCR_rev	AGTCAAGGTGCTTTTGGTGTAG
GA20ox2_1	Potri.002G151300	GA20ox2-1_qPCR_fwd	CGAAAAACCATGCCTTGAATC

GA20ox2_1	Potri.002G151300	GA20ox2-1_qPCR_rev	GCCAAAGGATCTCCAGTGAG
GA20ox3	Potri.005G184400	GA20ox3_qPCR_fwd	CTTCGGATCTCGTTGTGCTAG
GA20ox3	Potri.005G184400	GA20ox3_qPCR_rev	CCAATATGGCAAAGGATAAATC
GA20ox5	Potri.007G103800	GA20ox5_qPCR_fwd	GAGCAGTTGCAACCTCATCA
GA20ox5	Potri.007G103800	GA20ox5_qPCR_rev	ACTTGCCCACAGAGTTCATG
GA20ox6	Potri.012G132400	GA20ox6_qPCR_fwd	CCAATTTCGACGCTTTTGTCG
GA20ox6	Potri.012G132400	GA20ox6_qPCR_rev	AAGCAAGAGATTTTCTTGGCG
GA20ox8	Potri.015G134600	GA20ox8_qPCR_fwd	TGGTGTCGAAGAACTTGTGC
GA20ox8	Potri.015G134600	GA20ox8_qPCR_rev	CATCAAAACCATGCCATCC
LAP1	Potri.008G098500	LAP1_qPCR_fwd	ATGCCGAGGTTGCCTTGATC
LAP1	Potri.008G098500	LAP1_qPCR_rev	GAATACCTCTCATGGCGTTCGAG
AIL1	Potri.002G114800	AIL1_qPCR_fwd	CTGGAATCAGTTATGGAGTCGGAG
AIL1	Potri.002G114800	AIL1_qPCR_rev	GGTTGTGATCTAGTGAGAGCTTCC
GID1A1	Potri.005G040600	GID1A1_qPCR_fwd	ACCGTGGGACTAGCCTTCTT
GID1A1	Potri.005G040600	GID1A1_qPCR_rev	ACAACGTCCGAGTTGACAGGC
GID1A2	Potri.013G028700	GID1A2_qPCR_fwd	GGACCGAGATTGGTACTGGA
GID1A2	Potri.013G028700	GID1A2_qPCR_rev	TAAACCAGCCACCACAACAA
GID1B1	Potri.014G135900	GID1B1_qPCR_fwd	GATCATGTTGATCGCACCAC
GID1B1	Potri.014G135900	GID1B1_qPCR_rev	GTGCTCAAGGGCTTTTCAAGC
GID1B2	Potri.002G213100	GID1B2_qPCR_fwd	GAGAGGGCCAGTTCCGG
GID1B2	Potri.002G213100	GID1B2_qPCR_rev	ACCTCCCCTAGCACTGTGG
35S promoter		35Sfwd	CTATCCTTCGCAAGACCCTTC
Cas9		Cas9rev	TTCCTCAGATGATATATGGTTGGG

Table S2. Values of GA_{19}, GA_{20}, GA_1, and GA_4 quantification in leaf and shoot apex of *ft-8* and WT obtained for three biological replicates.

Tissue	Genotype	GA_{19} (ng/g)	GA_{20} (ng/g)	GA_1 (ng/g)	GA_4 (ng/g)
Shoot apex	WT	12,76	0,46	9,98	0,23
Shoot apex	WT	13,64	0,49	8,88	0,14
Shoot apex	WT	15,55	0,60	8,52	0,15
Shoot apex	*ft2-8*	7,57	0,19	5,46	0,23
Shoot apex	*ft2-8*	6,31	0,11	8,29	0,15
Shoot apex	*ft2-8*	5,19	0,14	3,47	0,16
Leaves	WT	1,38	0,51	2,65	0,10
Leaves	WT	1,68	0,35	1,72	0,11
Leaves	WT	2,72	0,41	1,46	0,11
Leaves	*ft2-8*	0,67	0,30	4,26	0,07
Leaves	*ft2-8*	0,39	0,30	5,62	0,10
Leaves	*ft2-8*	1,05	0,34	5,52	0,07
